# Key strategies to improve systems for managing patient complaints within health facilities – what can we learn from the existing literature?

**DOI:** 10.1080/16549716.2018.1458938

**Published:** 2018-04-16

**Authors:** Tolib Mirzoev, Sumit Kane

**Affiliations:** a Nuffield Centre for International Health and Development, University of Leeds, Leeds, UK; b Nossal Institute for Global Health, Melbourne School of Population and Global Health, University of Melbourne, Melbourne, Australia

**Keywords:** Patient feedback, health system strengthening, accountability, responsiveness

## Abstract

**Background**: Information from patient complaints – a widely accepted measure of patient satisfaction with services – can inform improvements in service quality, and contribute towards overall health systems performance. While analyses of data from patient complaints received much emphasis, there is limited published literature on key interventions to improve complaint management systems.

**Objectives**: The objectives are two-fold: first, to synthesise existing evidence and provide practical options to inform future policy and practice and, second, to identify key outstanding gaps in the existing literature to inform agenda for future research.

**Methods**: We report results of review of the existing literature. Peer-reviewed published literature was searched in OVID Medline, OVID Global Health and PubMed. In addition, relevant citations from the reviewed articles were followed up, and we also report grey literature from the UK and the Netherlands.

**Results**: Effective interventions can improve collection of complaints (e.g. establishing easy-to-use channels and raising patients’ awareness of these), analysis of complaint data (e.g. creating structures and spaces for analysis and learning from complaints data), and subsequent action (e.g. timely feedback to complainants and integrating learning from complaints into service quality improvement). No one single measure can be sufficient, and any intervention to improve patient complaint management system must include different components, which need to be feasible, effective, scalable, and sustainable within local context.

**Conclusions**: Effective interventions to strengthen patient complaints systems need to be: comprehensive, integrated within existing systems, context-specific and cognizant of the information asymmetry and the unequal power relations between the key actors. Four gaps in the published literature represent an agenda for future research: limited understanding of contexts of effective interventions, absence of system-wide approaches, lack of evidence from low- and middle-income countries and absence of focused empirical assessments of behaviour of staff who manage patient complaints.

## Background

Information from patient complaints – a widely accepted measure of patient–provider relationships and patients’ satisfaction with services they receive – can contribute towards improved patients’ engagement in health services [1], improved quality of health services [2–11], health staff review, management and development [10,12–18], improved accountability [19–23], reduced abuse, assured compliance with standards and improved overall health systems performance [2,24]. Therefore, effective patient complaint management systems constitute a crucial component of well-performing national health systems.

An effective patient complaints management system entails collecting and analysing complaints data, as well as acting upon this information. Two issues are central to effective system of managing patient complaints. First, is the opportunity for patients to provide feedback on their experiences (e.g. the care they receive, staff expertise and availability of supplies [3,25,26]) and to complain when their experiences do not align with their expectations. Second, is the ability of the health system to adequately analyse, respond to, and utilise patient feedback – for example in health service quality improvement (QI) [5,9,25] or improving human resource management processes (HRM) [9,18,27,28]. A key lesson from recent reviews is the need for patient complaints to be a part of an integrated system that ensures patient feedback is responded to [8,29,30] and always acted upon [8,9,25,31]. People who receive no response to their complaints can feel frustrated and disengage with health services or even worse, resort to violence, as is increasingly the case in some countries such as China or India [32,33].

There is substantial literature on the nature and amount of patient complaints. A recent systematic review identified a range of issues that inform patient complaints within the health sector [3]. Issues of *safety and quality of care* account for 33.7% of all complaints, *management-related issues* (processes related to admission, discharge, billing, finances, referrals) for 35.1%, and problems in *staff–patient relationships* (behaviour, conduct, communication) for 29.1%. Another systematic review showed that in most parts of the world, complaint rates are low when compared to preventable adverse events; that certain groups use available complaint procedures more than others; and that people are more likely to complain informally than formally [34]. A review of 5,375 patient records from 14 hospitals in the Netherlands compared preventable adverse events with informal and formal patient complaints, medico-legal claims by patients and incident reports by staff [35]. The authors found that only 3.6% of the adverse events identified through review of records was found in these reporting systems. Similarly, a telephone survey in Israel revealed that, while 25% of the respondents had a cause to complain only 9.5% actually complained, and of these most complained informally [36]. However, when patients do complain their reporting of adverse events has been shown to be generally reliable [37]. We argue that health systems have a responsibility for identifying such emerging patterns [10] by analysing such data, and using these results for improving systems performance.

There is a large body of work on typologies, empirical assessments of nature of patient complaints, and contributions of patient complaints to service quality improvement. However, there is limited published literature synthesising the effective interventions to improve patient complaint management systems, particularly from low- and middle-income countries (LMICs). In this paper, we attempt to bridge this gap. The objectives of this paper are two-fold: first, to synthesise existing evidence in order to provide practical options to inform future policy and practice and, second, to identify key outstanding gaps in the existing literature to inform agenda for future research. In achieving the first objective, this review seeks to answer the question: *what can we learn from the existing literature on effective strategies to improve systems for managing patient complaints?* To answer this question, we draw on both conceptual and empirical work, to provide a comprehensive overview of key interventions to strengthen patient complaint systems. In doing so, we utilise a three-step framework for classifying these interventions.

This review should be of interest and relevance to different readers: academics who are engaged in conceptualizing and assessing effectiveness of measures to improve patient complaint systems, and clinicians, managers and policymakers who are interested in improving patient complaint systems and ultimately strengthening wider responsiveness of their national health systems.

The paper is structured as follows. After describing our methodology, we provide an overview of effective interventions to strengthen patient complaint systems which is structured along the three key steps in the complaint management processes. We then summarise these strategies, and identify key outstanding gaps in the existing literature on this subject.

## Methods

This paper reports an overview of the literature on key strategies to strengthen patient complaint management systems. While this piece does not fit the parameters of a fully fledged systematic review with pre-established criteria for assessing data quality and checks by at least two researchers, the structured literature search in this overview of the literature can be described as a ‘systematic search and review of literature’ [38]. According to Grant and Booth, this review approach aims for a comprehensive search while not necessarily requiring assessment of quality of identified studies to determine inclusion or exclusion, thus combining ‘…*strengths of critical review with a comprehensive search process’* [38, p.94]. While the rigour and discipline of systematic reviews can usefully account for and mitigate potential bias and help assess the strength of existing evidence base, it requires far more time and resources than were available to us for this study. Thus, given the complete absence of published reviews on this topic, our intention here is to provide a timely overview, and practical recommendations to inform policy and practice of strengthening patient complaint systems within national health systems.

Peer reviewed published literature was searched in three databases: OVID MEDLINE(R) Versions, OVID Global Health (1973 to 2017 week 26) and PubMed. The literature search was conducted initially during January-March 2016 and was subsequently updated in June-July 2017. It was guided by the following keywords: patient*, citizen*, complain*, feedback*, strengthen*, improve*, strateg*. Following the searches using individual keywords, the search results were narrowed down using built-in filters within the databases: English-language literature; availability of abstract.

The search returns for individual keywords, narrowed down by review articles and the specified timeframe, resulted in about 4.6 million resources and eventually the different combinations of these keywords resulted in 486 resources identified for review. The titles of each of these papers were screened by one researcher for relevance to the topic of this review (i.e. interventions to strengthen patient complaint systems), and if found relevant then further selection was based on the reading of the abstracts and subsequently the full texts. Abstracts and subsequently full-texts, were included based on the combination of two criteria: existence of clear intervention to improve patient complaint system and identification of effects of the implemented intervention(s). Eventually, 72 papers were selected in this review (414 were excluded).

In addition, relevant citations from the reviewed articles were followed up; this resulted in a few more resources being included in the review (n = 7). We have also drawn on some grey literature which we were aware of from the UK and the Netherlands (n = 4), though we did not perform a structured search of grey literature. Finally, a search using a generic search engine (Google Scholar) was performed as an additional measure to ensure that no key resources were omitted. This helped to identify 58 further publications; of these, two were included in the review, and the remaining 56 were excluded because they were duplicates of papers selected from the earlier database search.

We report the results using a three-step framework for complaint management processes. These steps include: collection of complaints, analysis of complaints data, and action on the information, and are briefly set out in the next section.

## Results

Up to five steps can be distinguished in the processes of managing patient complaints within health facilities: receipt of complaint, classification of complaint by its type and nature, settlement, resolution, and closing and reporting [25,39]. From our analysis, we conceptually distinguish the following three broad steps in the patient complaint management processes:

1. *Collection of complaints*, which is contingent upon the existence of appropriate policy and regulatory framework, patient capacity, desire and willingness to complain (often determined by patient expectations of the complaints system), and availability and patient awareness of adequate and easy-to-use information collection tools (e.g. website repository, telephone hotline or suggestion boxes in health facilities);
2. *Analysis of complaints data*, which is determined by availability of appropriate structures (e.g. separate unit in hospitals) with skilled staff who are able to accurately analyse complaints, and effectively communicate results of this analysis to facility managers;
3. *Action on the information*, including: (a) resolving the issue and responding to the complainant (including reporting that appropriate action has been taken) and (b) using the information within health facilities, for example for service QI through integrating information into regular management reviews and other QI mechanisms.

All three steps are undoubtedly important and clearly interrelated. However, our analysis also points to three caveats. First, while audited and based on selected evidence, many effective best practices in improving complaints management systems are driven by practical experiences and only documented in the grey literature – they are seldom reported in the academic literature [3,40]. Although methodological rigour of grey literature can be legitimately questioned, we consider these as important sources of evidence which often have high impact on policy and practice. Second, evidence of effective interventions covering each of the three steps appears to be unevenly distributed, with most studies focusing on the first (collection of complaints) or the third (action on the information) steps. The ‘bit in the middle’ (i.e. analysis of complaints data) is often not directly reported in the literature, though there are many studies that allude to the effectiveness of interventions to improve analytical skills and expertise of staff. Third, literature highlighting effective interventions covers mostly specific interventions within each of these three steps, and the relationship between the three steps is often rather implicit.

We return to these caveats later in the paper, when discussing the opportunity these provide for future research. Next, we summarise existing evidence of effective interventions for each of the above three steps in the complaint management processes.

### Effective interventions to improve collection of complaints

Interventions to improve collection of complaints include two interrelated categories: those aimed at improving patients’ initial willingness to complain and those aimed at creating an appropriate policy and institutional framework to support staff to receive and adequately document complaints data.

Interventions to improve systems of patient complaints promote behaviour change of both patients (to enable them to complain, when appropriate) and providers (to enable them to effectively respond to complaints) [41]. While there is substantial literature on determinants of behaviour of health staff [42], we found no articles that focus exclusively on staff behaviour to manage patient complaints. As for behaviour of patients, three key drivers of behaviour – opportunity, capability and motivation [43], can be targeted to improve patients’ willingness to complain, as we discuss next.

In relation to *capability*, an individual’s capability to act, or their ‘agency’, is shaped by different social structures and social relations. Certain population groups (in terms of age, gender, ethnicity) have been found to complain less than other groups; and some have more opportunities (social, political, economic, etc.) and resources to exercise their agency than others [44]. Different outreach-based interventions (e.g. community-based data collection through a survey or a toll-free hotline) have been shown to effectively reach vulnerable and disadvantaged groups [36,45–50]. Studies also show that when individuals are unwell, their usual capability may be suspended, and family members or others need to make complaints on their behalf [2,45].

Evidence also shows the effectiveness of interventions for promoting awareness amongst citizens (including patients and their families) about their rights and helping them to demand accountability through exercising their rights. These interventions include both supply- (i.e. focusing on health facilities) and demand-side (i.e. focusing on patients and their families) interventions such as appointing dedicated complaints officer or confidential counsellor positions, making the complaints procedure user-friendly for patients, introducing citizen monitors, and catalysing and resourcing the formation of patient groups [36,45–49,51–56]. For example, in India, awareness raising combined with a toll-free hotline where women could report demands from service providers for informal payments have ‘*enhanced women’s knowledge of their entitlements, as well as their confidence to claim their rights*’ [50, p.E135] and in Peru citizen monitoring by indigenous women has shown to improve identification, documenting, and action on ‘*everyday injustices*’ thus leading to important changes at the health facility level [56].

*Opportunity* refers to possibilities and spaces for citizens to easily express or communicate their complaints. These can be at the community level, at the interface of services and communities, within the services, and in spaces specially created for the purpose. Globally, more patients complain informally than formally; this either signals poor access to formal complaints processes and/or that complaints made through formal channels are not acted upon [15,36]. Evidence highlights the importance of improvements in complaints management, through creating opportunities and spaces within health systems for citizens to get redressal for their grievances to complement demand side-only interventions [51,52].

Evidence also shows that increasing patient awareness of the existing complaints channels, and patient rights, can be effective in improving their ability to complain, as demonstrated in Sweden and Finland [31,57]. Raising awareness about, the options available, the ease of access to different options, the results that each option offers, the fairness of the process and the commitment to justice, should enhance citizens’ ability to complain [58].

Using different ways of obtaining feedback from patients on their experiences of health care can provide opportunities and spaces for, and also increase the capture of, patient complaints [7]. Different systems can complementarily co-exist within a single context; for example in Bangladesh a health sector-specific patient feedback SMS-texting system [59] and health ministry’s call centre [60] are supplemented by a wider government level grievance redressal system [61]. A user-friendly complaints reporting system, for example, using a telephone-based process [45], has been shown to improve access to complaints processes. A review of 17 publications from different countries found that context-specific ways of eliciting information from service users (e.g. individual interviewing of hospitalised patients or conducting follow-up by telephone on patient experiences of using health services) can be most effective in increasing the amount of patient feedback [37]. Other studies have also shown the effectiveness of actively solicited patient feedback [37,62].

With regards to *motivation*, research shows that patients are more likely to express their complaints if they feel that justice will be done and an improvement in the quality of care will result from their complaint [2,58,63]. Communication with patients about actions taken is therefore especially important, as is involving patients in the complaints handling process itself. Studies have highlighted the effectiveness of better hospital partnership with patient advocacy organisations, as key to an effective and learning-oriented complaints system [7,55]. As one systematic review highlighted, over two-thirds of all complaints involve dissatisfaction with human interaction [3]. Therefore, any measures that involve learning from complaints for QI should draw on the participation, co-operation and initiatives of doctors, nurses and other staff [64]. Other authors have argued for ‘*more emphasis… on the quality of interpersonal interaction… for successful resolution of complaints. Attending to the process alone will not reduce dissatisfaction…*’ [65, p.164]. Inevitably, any quality improvement process involves organisational change, and in the context of complaints management processes, it requires a major human resource management element.

Evidence shows that *appropriate policy, regulatory framework and regulatory authority*, are all critical to enhancing access to, and improving collection of, complaints. A systematic review published in 2014 [34] highlighted the importance of strategic and responsive approaches to health complaints governance, including ‘*networked governance and flexible or responsive regulation*’, and the need to better understand how best to collect and harness complaints data to systematically improve service quality. The authors also argued for the need to examine the processes of complaints in health and social system contexts to make these processes fairer and better able to meet the complex needs of complainants, health professionals and society [34].

In the Netherlands, patient complaints are addressed within a comprehensive legislative framework laid down in the Healthcare Quality, Complaints and Disputes Act (WKKGZ) of 2016 [65]. The WKKGZ builds on and combines two different acts, namely the Quality of Health Services Act (1991) and the Healthcare complaints Act (1994) to further strengthen patient rights in a single piece of legislation.

The existence of an independent Ombudsman in Finland [57], a quasi-independent body such as a Patient Advisory Committee in Sweden [31], or the Health Care Inspectorate in the Netherlands [63], have been shown to make the complaint-handling processes more independent and to improve the monitoring of such processes, particularly when supplemented by local arrangements to operationalise and implement the policies. Examples of such effective local arrangements include Local Medical Committees and the Patient Advice and Liaison Service in the UK, and independent dispute committees and patient councils in the Netherlands. Health systems in low- and middle-income countries (LMICs) often have broad policy and regulatory frameworks and national bodies for complaints handling, but they struggle with operationalising and implementing these policies [9,25]. Experiences of different high-income countries in operationalising their policies can be relevant to health systems in different LMICs, such as in Vietnam where public hospitals face an increased need to maintain their reputations to maintain their patient numbers and thus ensure their financial sustainability [25,66] or in China and India where failures to effectively redress patient grievances are observed amidst alarming rise in violence towards health staff [32,33,67].

A clear set of principles which can serve as a framework to guide the involvement of different actors in the complaints management processes, is thus important. In the UK, the Parliamentary and Health Service Ombudsman developed, based on their 40 years’ experience, six key principles for good complaints handling – get it right; be customer-focused; be open and accountable; act fairly; put things right; and seek continuous improvement [68–70] – which apply to all public sector actors. Table 1 shows different actors’ roles in the complaints processes in the NHS England compared with those in the Netherlands.

**Table 1.** Key actors and their roles in patient complaints in NHS England and the Netherlands [71,72].

NHS England	The Netherlands
**Local NHS Service Provider (GP Practices, Hospitals, NHS Trusts)**• First point of contact, with Named Responsible Person and Complaints Manager• Clear procedures for complaint handling locally, compliant with NHS requirements**NHS Commissioner (NHS England or Clinical Commissioning Group)**• Received complaints usually first passed to local GP Practice, Hospital/NHS Trust if not already done• NHS England can take on investigation itself if clear criteria are met• Complaints Managers provide support to NHS staff responsible for handling locally**Parliamentary and Health Service Ombudsman (Independent body)**• Publish the NHS principles, guidelines, frameworks, from 40-year experience• Manage complaints if local processes failed, but legal criteria must be met• Share information with CQC, regulators, local NHS Trusts, policymakers, patients**Local Medical Committees (LMCs, local representative committee of GPs)**• GPs can inform LMC if they feel the system is being abused, or they are being treated unfairly by NHS**Patient Advice and Liaison Service (PALS, groups associated with NHS England and NHS Trusts)**• Support to patients with grievances, who may not wish to make a formal complaint• Information to support patients in making choices about health and care services**Care Quality Commission (CQC)**• Inspect and monitor health and care services **Healthcare professional regulators (e.g. GMC, NMC)**• Regulate staff professional standards **Citizens Advice Bureau**• Guidance for, and information on organisations that help, to make complaints against health services, including taking complaints to court	**All healthcare providers (except those covered by Social Support Act)**• First point of contact, Complaints Officer and Confidential Advisor• Clear procedures compliant with the WKKGZ (new law, in effect since 1 January 2017).**Disputes Committee (Independent body)**• An accessible way to receive a binding ruling• The WKKGZ stipulates that all care institutions join a recognised disputes committee/commission.• Clear procedures for complaint handling• Patient organisations can also use this procedure• All providers need to affiliate with a disputes committee **Patients’ Council**• Support to patients with complaints or grievances.• Advise management on decisions that affect patients (eg. quality of care, residential issues)• Facilities to provide resources for these councils.• Can influence management and supervisory boards**The Health Care Inspectorate (IGZ)**• Oversees WKKGZ implementation through guidelines.• Does not deal with complaints from individuals, but inspects and monitors health services.• They investigate if complaints point to structural issues; IGZ can also file complaints• They share information with other regulators, management boards, and patient organisations.**Medical Disciplinary Tribunals(Tuchtcolleges)**• Regulating standards of healthcare professions.• Medico-juridical boards, who can issue warnings, fines, or may prohibit a professional from practice.**Dutch Centre for Consumer Experience in Health Care**• An independent foundation representing patients, insurers, and providers• Inspection and monitoring of health and services**Support and guidance for patients making complaints (examples)**• Federation of Patients and Consumer Organisations• Dutch Patient Association• The National Health Care Report Centre**Civil Courts**• Further advice to those not satisfied with the grievance redressal process• Required for those seeking financial compensation for amounts greater than 5000 EUR.

Appropriate policy and institutional frameworks are significant determinants of staff motivation, behaviour and performance [27,30,42,73]. While there is lack of studies which explore behaviours of service providers in managing patient complaints, a systematic review of factors that influence the self-reporting of incidents by health staff identified that *‘fear of punishment, uncertainty of what should be reported and how incident reports will be used and time constraints to incident reporting are common barriers to incident recognition and reporting’* [74]. Similar findings were reported from a more recent systematic review of critical incident reporting system conducted by Health Quality Ontario in Canada [75]. Within the context of patient complaints, this evidence highlights the importance of training front-line staff in complaints handling, including, on how to deal with patient emotion or aggression associated with complaints, and in recognising possible patterns and ‘domino effects’ (index complaints may represent a series of previously un-reported grievances) [15,16]. Receiving complaints can also be a difficult experience for individual providers or facilities, and may affect subsequent behaviour, for example over- or under-investigation and response [17]. Related to staff training, patients emphasise staff communication as being important to them; nature and quality of provider communication, including during the process of addressing grievances are highlighted in the literature as a common factor in patient complaints; effective communication is central to a complaints redressal process. Therefore, training of staff in effective communication is an essential part of effective complaints management systems [76–79].

### Effective interventions to improve analysis of complaint data

Research shows that appropriate registration of complaints within health facilities, for example through using a standardised taxonomy [80], can improve the quality of analysis of patient complaints. A common database and systematic approach to recording, helps avoid complaints being viewed as one-off incidents. It allows data to be examined for patterns, and to form the basis for service QI [5,7,10,16].

Standardised templates for phone calls and emails related to the patient complaints process were shown to improve the complaints management and analysis procedure in the United States (US) [7]. In Bangladesh, service users can text their complaints to a mobile number displayed on information boards in public health centres [81]. Staff at the health ministry monitors all texts displayed through a publicly accessible web portal (http://app.dghs.gov.bd/complaintbox/), and follows-up arising issues with both the sender and local authorities thus prompting the analyses and actions on complaints [59]. Further effective interventions to improve analysis of complaints in health facilities included:

- Registration of complaints by dedicated officers [6,17]
- Existence of risk management teams within health facilities [17]
- Creation of patient relations departments with capacity to effectively communicate with the public [7,53]
- Establishment of a separate post of hospital mediator (e.g. chief of staff) where appropriate and feasible [82]
- Creation of structures and spaces for intra-organisational learning from complaints [7,83,84]

Analysis of complaints can identify particular staff members who may benefit from support. Studies have found that patient complaints about individual staff are skewed. For example, in a US study of urologists, 47% had no complaints, while 11% were associated with 50% of the complaints [85]. Other studies also found similar patterns [10,13,14]

Availability of appropriately trained staff is a crucial component of effective complaint management system [7]. In the UK, NHS Scotland included training in handling complaints as part of staff induction and performance appraisal, as a measure of ensuring that all staff had sufficient knowledge and skills in relation to complaint handling. A study comparing different approaches to taking patients’ voices into account within quality management systems in Taiwan showed that existence of dedicated QI officers to handle complaints can be particularly effective in ensuring the integration of information from patient complaints into service QI [6].

### Effective interventions to improve action on complaints data

Two groups of effective interventions to improve action on complaints can be distinguished in the literature: mutually acceptable resolution with patients, and improving use of complaints data in service quality improvement (QI) and human resource management (HRM).

Adequate response to each complaint is an essential attribute of effective complaint management systems [86]. Evidence from the Netherlands shows that for complainants, an assurance that their complaint will lead to improvements and change is as important as personal redressal [87]; and that an honest commitment to learning from mistakes, at provider, organisational, and policy levels, is appreciated by patients, and will enhance accountability and improve the care experience [88]. Meanwhile, studies in the UK showed that over half of complaints can be effectively and quickly settled with an apology or an explanation [89] or by a single telephone call or letter-based response [40]. In Vietnam, public hospitals also quickly resolve complaints. This, however, appears to be so because these semi-autonomous facilities need to maintain their reputation (and consequently patient numbers) within a decentralised context [25]; therefore, the quick resolution appears to be often at the expense of learning and change within health facilities.

The literature on integration of patient complaints within wider health systems emphasises use of complaints data in two health systems components: a) service QI and b) HRM. Each component, being amongst the six building blocks of health systems [90], is crucial to well-functioning and responsive health systems [91–93]. The effects on these two components are often related – for example, improved use of complaints data in mental health hospitals in England was shown to lead to improvements in treatment programmes, staff shortages, and quality of meals [94].

In the Netherlands, where in 2016 the health system was ranked 1st by the Eurohealth Consumer Index report for the fourth year in succession, there is structured participation of patients in health decision-making [55], and learning from complaints handling occurs at multiple levels i.e. provider, organisation and policy [95]. A gap between patient expectations and actual improvements in quality of care as a result of a complaint was highlighted in the Netherlands, where patients received little feedback on any changes resulting from their complaints [2]. In response, the recent improvements in the Dutch Healthcare Inspectorate’s (IGZ) oversight of complaints processes now entail more active involvement of patients in the complaints process as a means to learn and ensure accountability, so that IGZ and health providers can capitalise on existing opportunities [63].

Patient complaints can be seen as a group of service QI issues, similar to confidential error or clinical incident reporting, medicine manufacturing, licensing of facilities and registration of health professionals – all of which are covered by legislation and are subject to statutory inspections in most European countries [96]. Patient complaint structures can also be usefully seen as being part of the institutionalisation of QI processes, and thus should be publicised and be universally accessible. The principles underlying such a view relate to the responsibility of healthcare providers and the health system to be responsive to patient’s needs, and to respectful of patient’s right to autonomy and dignity [97,98]. Health service QI interventions resulting from patient complaints data can occur at unit and facility [94], as well as systems levels. Research also shows that QI interventions in turn can make the overall complaints procedure more cost-effective [99].

Complaints data analysis is reported as a method of service QI and a measure of service quality [7–11,96,100,101], which requires ‘*an effective bridge, at a national policy level, between the patient complaints management system and the quality management system*’ [9 p.91]. Most health service QI approaches consider perspectives of: patients, public, professional and managerial [96,102]. Well-designed complaint resolution processes provide opportunities to develop appropriate strategies leading to greater patient satisfaction [3,7,64,103–107]. Improved understanding of the reasons for, and nature of, complaints can provide insights into delivery of particularly complex care, detect lapses that providers may have missed, and address these issues before they escalate into substantial problems [8,11,30,40,53]. However, these opportunities can often be missed, because the aim may be to avoid litigation or reputational damage as was found in England and Vietnam respectively [25,53], because of lack of blame-free culture that encourages reporting and learning [75], or because QI interventions are implemented at a single unit level [94] thus preventing systems learning [8,9,94].

A recent systematic review provides a useful starting point for HRM interventions that can incorporate learning from patient complaints [3]. Effective interventions include various approaches (e.g. training, group and self-reflection sessions, mentoring and coaching) to improve the communicatory aspects of relationships between patients and staff in order to develop, improve and sustain components of health systems responsiveness (e.g. humaneness, sensitivity, empathy, caring nature, respect and dignified interaction, amongst various cadres) [3,98,108,109]. This is consistent with sociological literature [4,110,111] which points to the centrality of learning from patient complaints, and to challenges involved in balancing systemic and management aspects and staff–patient relationships. Critically examining and then addressing the tensions between ‘relationships’ and ‘system’ issues within healthcare organisations can also be a useful approach for understanding and learning from complaints [112].

For example, implementation of a peer-support programme was proven to be effective in supporting clinicians who had been identified as receivers of complaints [7]. This was done in recognition that complaints are often linked to specific physicians. Complaints data can therefore be harnessed by leaders to identify staff receiving a disproportionate number of complaints, to offer specific support and training [99,113].

Similarly, a study conducted in an Israeli hospital identified that data from patient complaints can inform effective interventions combining three groups of resources: human resources (skills and numbers), technological resources (e.g. introduction of ambulatory examinations under sedation for children) and procedures (e.g. organising a flexible committee to review and respond to complaints within 2 weeks of receipt) [64].

## Discussion

In this paper, we examine the key strategies to strengthen patient complaint management systems in our attempt to propose practical recommendations for future policy and practice.

There is substantial evidence of effective interventions to improve each of the three steps of patient complaints management processes originating from high-income contexts whereas published knowledge from LMICs is limited. Although studies often report interventions within each step, we argue that many interventions can effectively address all three steps of the complaint process as shown in Table 2.

**Table 2.** Summary of effective interventions to improve systems of patient complaints.

Collection of complaints	Analysis of complaint data	Action on complaints
Raise public awareness of rights and available complaint channels [31,50,57] Provide dedicated complaints officer, confidential counsellors, patient groups and citizen monitors [36,45–49,54–56] Train staff on how to deal with patient emotion, recognise patterns and communicate effectively [15,16,76–78] Implement user-friendly system of soliciting feedback e.g. telephone, interviewing and follow-up [37,45,53,62] Carry out outreach to reach vulnerable groups [36,45–49]	Instigate a common database and systematic approach to recording to find patterns [5,7,10,16] Develop standardised templates for calls and emails [7] Identify dedicated officers to register complaints [6,17] Create separate patient relations department [7,53] or mediator [82] Develop structures and spaces for learning within organisations [7,83,84]	Ensure adequate and timely response to each complaint [3,7,86], even if a simple apology or an explanation [40,89] Ensure effective communication through training, reflection, mentoring and coaching [3,4,79,110,111] Encourage QI at unit or facility levels [94] Provide peer-support and training to receivers of complaints [7,99,113] Instigate centralised reporting [7]
Develop robust policy and regulatory framework [34,68–72,95] Appoint independent regulator and mediator e.g. ombudsman, committee or inspectorate [31,57,63] Establish risk management team within health facilities [17] Implement demand-side interventions (e.g. awareness raising) to enhance social accountability [51,52] Create partnerships between hospitals and patient advocacy organisations, for example through implementation of hospital governing boards [7,55,64,114–116]		

Interventions to improve systems of patient complaints, ultimately, should modify existing behaviours [41,43] of patients (enabling them to give feedback and to use the complaints channels where necessary) and staff (to effectively respond to, and act upon, the complaints). These, in turn, can lead to significant reductions in numbers of subsequent complaints [12]. A well-designed complaints capture and analysis process is likely to increase the number of complaints in the short-term, leading to improved service quality [7], and eventually to reduction in the complaint numbers in the longer-term. Centralised identification of issues arising from complaints at the individual, unit, procedural or organisational levels [64] can help to plan appropriate interventions [3,7,64,103–107]. However, complaints should ideally be dealt with locally, to ensure speedy and timely responses and avoid complex response processes [105].

Evidence also indicates that no one single measure will be sufficient to improve a patient complaint management system. For example, evidence shows that a combination of awareness raising and toll-free hotline improved women’s knowledge of their rights and increased their confidence to exercise these in India [50], and a combination of leadership engagement, centralised reporting, and improving response times was required to improve complaint systems in the US [7]. Therefore, any intervention to improve the patient complaints system must include different components, which of course need to be feasible, effective, scalable, and sustainable within that specific context.

The existing literature suggests that effective interventions to improve and integrate patient complaints within wider health systems need to be:

- *Comprehensive* i.e. comprising different components (e.g. regulatory measures complemented with staff training and awareness-raising of the public) covering all three steps in the complaints process which should together form a consistent package;
- *Integrated* within existing systems i.e. taking advantage of current systems and processes of particularly QI and HRM;
- *Context-specific* i.e. feasible within the current context of the national health system with its management styles, organisational culture, rules and cadre profile and numbers;
- *Cognizant of the information asymmetry and the unequal power relations* between the patients on one hand, and the professionals and the bureaucracies of service provision on the other; while at the same time, capable of navigating and shaping for the better, the complex professional, managerial and hierarchical interpersonal power relations existing between the different health systems actors.

We have shown that different interventions exist to improve each of the three steps in complaint handling processes. Evidence of effective interventions often comprises practical experiences and is within grey literature, which we consider as legitimate and important sources of evidence having high impact in informing policy and practice. Literature covering each of these three steps is unevenly distributed, with most studies focusing on the first or the third step of the complaint management process. Furthermore, evidence on effective interventions covers mostly interventions within a single step, rather than bridging the three key steps.

Finally, our review identified four gaps in the published literature, which in our view constitute an agenda for future research on this topic. *First, limited understanding of contexts of effective interventions*. There is a growing recognition of the importance of the context in ensuring the success of complex health systems interventions [41,117–122], including improvements to patient complaints systems. While specific components of the interventions are usually well described, it is often unclear which contextual facilitators and barriers affected their effectiveness, and how. There is therefore a major knowledge gap on the broader social, cultural and political culture, and if and how it shapes both citizens’ complaining behaviours, and bureaucracies’ responses to these complaints. In the same vein there is a knowledge gap about the organisational and bureaucratic cultures of public and private health services, and if and how it shapes both citizen’s complaining behaviours, and bureaucracies’ response to and learning from these complaints.

*Second, lack of health systems-wide approaches*. Many interventions to improve patient complaints, perhaps understandably, focus on just one of the three steps of the complaints process. Furthermore, effects of strengthened complaint management are mostly assessed in relation to a specific component of the wider health system, most notably health service quality. We found no literature on the integration of patient complaints systems, covering all three stages of the complaint process, within the wider health system. Beyond these knowledge gaps, in terms of policy and practice, this review highlights the need for patient complaint handling systems to be strengthened and better embedded within the health system. An explicit orientation towards system level learning from patient’s complaints can help improve the care experience for patients, and contribute to health system’s achieving their goal of being responsive [98].

*Third, lack of evidence from LMICs*. There is extensive literature, albeit insufficiently contextualised, on complaints handling in healthcare settings from high-income settings, most notably Europe. However, there is little research reported from LMICs, raising the need for more research on this topic from low resource settings. This may also reflect a widespread anecdotal experience of limited patient involvement in health care and a lack of integration of information from patient complaints, in service quality improvement in low-income country settings. From our review, we would especially call for greater reporting of effective interventions to improve actions on complaints data.

*Fourth, absence of empirical assessments of behaviour of staff who manage patient complaints*. As we have shown earlier, there is substantial literature examining patients’ behaviour. There is also extensive published knowledge on key determinants of staff behaviour, in particular motivation and performance. However, we found no empirical work that examines the behaviour (and its antecedents) of service providers and other staff involved in managing patient complaints, particularly from LMIC contexts.

### Study limitations

We acknowledge three limitations of our study. First, the overview of the literature reported here does not fit the parameters of a systematic review. Given the absence of literature on this topic, our intention was to provide timely and useful recommendations to inform policy and practice and inform future debate on key strategies to strengthen patient complaint systems. We recognise, however, that a fully fledged systematic review can be an agenda for future research on this topic. Second, although we believe in the comprehensive nature of our literature search, other studies on this topic can exist beyond the three databases covered in our review. In particular, although a more comprehensive search of the grey literature would be particularly beneficial to identify the unpublished guidelines and practices. Third, our search was limited to English language only and deploying multi-lingual teams can bring further useful resources to the review available in other languages (see e.g [86]).

## Conclusions

In this paper, we reviewed literature on key strategies to improve patient complaint management systems. There is substantial evidence of effective interventions to improve each of the three steps of patient complaints processes: collection of complaints, analysis of complaints and action on complaints information. Although studies often reported interventions within each step, we argue that many interventions can effectively address all three steps of the complaint process. Our review suggests that effective interventions to improve and integrate patient complaints within wider health systems should be: comprehensive, integrated within existing systems, context-specific and cognizant of the information asymmetry and the unequal power relations between patients, and the professionals and the bureaucracies of service provision. We identify four gaps in the published literature, which constitute agenda for future research on this topic: limited understanding of contexts of effective interventions, lack of system-wide approaches, lack of evidence from LMICs, and absence of empirical assessments of behaviour of staff who manage patient complaints.

## Paper context

Information from patient complaints is important for improving health systems performance. We report synthesis of literature on effective interventions to improve complaint management systems, and provide practical options for policy and practice. Interventions can improve collection of complaints, analysis of complaint data, and subsequent action on the information. Effective interventions to strengthen patient complaints systems need to be: comprehensive, integrated within existing health system, context-specific and cognizant of unequal power relations between the key actors.
